# Maximum acceleration performance of professional soccer players in linear sprints: Is there a direct connection with change-of-direction ability?

**DOI:** 10.1371/journal.pone.0216806

**Published:** 2019-05-14

**Authors:** Irineu Loturco, Lucas A. Pereira, Tomás T. Freitas, Pedro E. Alcaraz, Vinicius Zanetti, Chris Bishop, Ian Jeffreys

**Affiliations:** 1 NAR–Nucleus of High Performance in Sport, São Paulo, SP, Brazil; 2 Department of Human Movement Sciences, Federal University of São Paulo, Santos, SP, Brazil; 3 University of South Wales, Pontypridd, Wales, United Kingdom; 4 Research Center for High Performance Sport—Catholic University of Murcia, Murcia, Spain; 5 Faculty of Sport Sciences—Catholic University of Murcia, Murcia, Spain; 6 Red Bull Brazil Football, Jarinu, SP, Brazil; 7 Faculty of Science and Technology, London Sports Institute, Middlesex University, London, United Kingdom; James Cook University College of Healthcare Sciences, BRAZIL

## Abstract

The purpose of this study was to examine the selective influences of the maximum acceleration capability on change of direction (COD) speed, COD deficit, linear sprint speed, sprint momentum, and loaded and unloaded vertical jump performances in forty-nine male professional soccer players (24.3 ± 4.2 years; 75.4 ± 5.4 kg; 177.9 ± 6.4 cm). Soccer players performed the assessments in the following order: 1) squat and countermovement jumps; 2) 20-m sprinting speed test; 3) Zigzag COD ability test; and 4) bar-power outputs in the jump squat exercise. Athletes were divided, using a median split analysis, into two different groups according to their maximum acceleration rates from zero to 5-m (e.g., higher and lower ACC 0-5-m). Magnitude-based inference was used to compare the differences in the physical test results between “higher” and “lower” acceleration groups. A selective influence of the maximum acceleration ability on speed-power tests was observed, as the higher acceleration group demonstrated likely to almost certain higher performances than the lower acceleration group in all measurements (effect sizes varying from 0.66 [for sprint momentum in 20-m] to 2.39 [for sprint velocity in 5-m]). Conversely, the higher acceleration group demonstrated a higher COD deficit when compared to the lower acceleration group (ES = 0.55). This indicates compromised efficiency to perform COD maneuvers in this group of players. In summary, it was observed that soccer players with higher maximum acceleration rates are equally able to jump higher, sprint faster (over short distances), and achieve higher COD velocities than their slower counterparts. However, they appear to be less efficient at changing direction, which may be related to their reduced ability to deal with greater entry and exit velocities, or counterbalance the associated mechanical consequences (i.e., greater inertia) of being faster and more powerful.

## Introduction

Modern soccer can be characterized by a progressive and substantial increase in game speed [1–3]. A growing body of evidence has shown that, in the main soccer leagues around the world, ball speed, sprint distance, and number of maximum sprints per match have been gradually increasing in recent years [1–3]. Notably, the vast majority of these high-intensity activities (e.g., very-short sprints) occur during decisive moments, such as contests for the ball, offensive or defensive actions, and goal-scoring opportunities [4–7], thus having the potential to significantly affect game outcomes. Therefore, coaches and researchers are constantly looking for better and more effective training methods to both improve and optimize the acceleration capability of professional soccer players.

In every sport, the maximum acceleration rate occurs during the initial phases of sprinting [8–11], from stationary, low, or moderate speed starts, when athletes demonstrate the greatest changes in velocity. Specifically in soccer, players regularly initiate their all-out sprints from movements of moderate speeds [10,12,13], covering distances of approximately 6 m from ~25 to 60 times per game, depending on playing position, competitive level, and match period [1,14,15]. From these data, it is possible to conclude that the capacity to achieve higher velocities at very-short distances (e.g., ≤ 5-m) is a critical component of successful performances [1,2,4,15]. Indeed, a relevant study by Faude et al. [4] showed that straight sprinting is actually the most frequent action that precedes goal situations in soccer, sequentially followed by jumps, rotations, and rapid change-of-direction (COD) maneuvers. As such, a more comprehensive and multifaceted set of speed-related skills is clearly necessary to produce faster and more efficient soccer athletes [16].

As soccer is a multidirectional team-sport [17], players often have to accelerate in different directions, decelerate, re-accelerate, and execute successive changes of direction [10]. To better cope with match demands, soccer players must not only be able to sprint faster over linear courses, but also to rapidly decelerate and accelerate when changing direction [10,18]. “COD ability” may be defined as the ability to perform sudden directional changes [18], and has been described as a complex skill, underpinned by a myriad of distinct factors, such as straight speed, running (and approach-speed) technique, strength, power, and leg muscle qualities [19–21]. These physical and technical requirements usually vary according to the particular demands of a given task, such as the consecutive number of maneuvers and “cutting-angles” (e.g., 45 or 180° turns) [19–21]. Due to this multifaceted nature, it becomes essential to identify the more appropriate strategies for assessing and interpreting COD performance [22,23].

Recently, the “COD deficit” has been suggested as a suitable approach to evaluate COD efficiency in team sport athletes [16,23–25]. The COD deficit refers to the additional time that a directional change requires when compared to a straight sprint over an equal distance (e.g., 10-m time compared to 505-agility test time) [23] or difference in velocity between linear sprint and COD measurements of equal distances [25]. Current studies have revealed that faster and more powerful players from different sports are generally less efficient at changing direction [16,25,26]. For example, Loturco et al. [16] reported that higher linear speeds are not associated with superior COD performances in young soccer players. Furthermore, it has recently been demonstrated that faster athletes may present increased sprint momentum (hence, inertia), which may hamper their ability to execute successive accelerations and decelerations during COD maneuvers [20,26]. However, there is a lack of evidence regarding this issue in professional soccer players.

As maximum acceleration (i.e., 0-5-m) plays a crucial role in soccer performance [1,4,27], and soccer practitioners frequently focus on optimizing this capacity in their players [28,29], it would be interesting to investigate whether athletes with greater acceleration rates are able to accelerate, decelerate, and re-accelerate more quickly when changing direction. In addition, as neuromuscular abilities are normally interrelated in elite team-sport athletes [25,30–32], it would be relevant to analyze if faster players can perform better than their slower peers in more traditional speed-power measurements. The purpose of this study was to examine the selective influences [33,34] of the maximum acceleration capability on COD speed, COD deficit, linear sprint speed, sprint momentum, and loaded and unloaded vertical jump performances in professional soccer players. Based on our previous research and experience, we hypothesized that greater acceleration rates would be directly connected with greater speed and power outputs, but not necessarily related to COD efficiency (i.e., a lower COD deficit).

## Materials and methods

### Study design

This was a cross-sectional study, which analyzed the selective influences of the maximum acceleration ability on a series of speed-power related tests. Due to the training and assessment routines in the investigated clubs, all soccer players were already familiar with the testing procedures. The order of the assessments was as follows: 1) squat and countermovement jump tests (SJ and CMJ, respectively); 2) 20-m sprinting speed test; 3) COD ability test; and 4) bar-power outputs in the jump squat exercise. The tests were organized from the simplest and least physically demanding (unloaded vertical jumps) to the most physically demanding (e.g., sprinting and loaded jumps). Prior to the tests, the athletes performed standardized warm-up protocols including general (i.e., running at a moderate pace for 5-min followed by active lower limb stretching for 3-min) and specific exercises (submaximal attempts at tested exercises). All tests were performed on the first day of the preseason. Between each test, a 15-min interval was allowed, to explain the following procedures and adjust the equipment. The physical tests were all performed between 9:00 a.m. and 13:00 p.m.

### Participants

Forty-nine male professional soccer players (24.3 ± 4.2 years; 75.4 ± 5.4 kg; 177.9 ± 6.4 cm) participated in this study. Participants were members of two distinct soccer clubs and were undertaking different training routines, as planned by their technical staff. The athletes in this manuscript gave written informed consent, as outlined in the PLOS consent form, to publish this study. This study was performed in accordance with the ethical standards of the Helsinki Declaration and was approved by the Anhanguera-Bandeirante University Ethics Committee.

### Vertical jumping ability

Vertical jumping ability was assessed using the SJ and CMJ. In the SJ, a static position with a 90° knee flexion angle was maintained for 2-s before a jump attempt without any preparatory movement. In the CMJ, the soccer players were instructed to perform a downward movement followed by a complete extension of the lower limbs and freely determine the amplitude of the countermovement to avoid changes in the jumping coordination pattern. All jumps were executed with the hands on the hips. Five attempts at each jump were performed interspersed by 15-s intervals. The jumps were performed on a contact platform (Elite Jump, S2 Sports, São Paulo, Brazil). The best attempt was used for data analysis purposes.

### Sprinting and acceleration abilities

Prior to the execution of the speed tests, four pairs of photocells (Smart Speed, Fusion Equipment, AUS) were positioned at distances of 0-, 5-, 10-, and 20-m along the course. The soccer players sprinted twice, starting from a standing position 0.3-m behind the start line. In order to avoid weather influences, the sprint tests were performed on an indoor running track. Sprint velocity (VEL) was calculated as the distance traveled over a measured time interval. The acceleration (ACC) capacity in the 0-5-m was calculated as the rate of change of velocity with respect to time. Sprint momentum (kg^.^m^.^s^-1^) was obtained by multiplying the athlete’s body mass by the respective velocities (5-, 10-, and 20-m) during the linear sprints [26]. A 5-min rest interval was allowed between the two attempts and the fastest time was retained for the analysis.

### Change of direction ability and deficit

**Fig 1:**
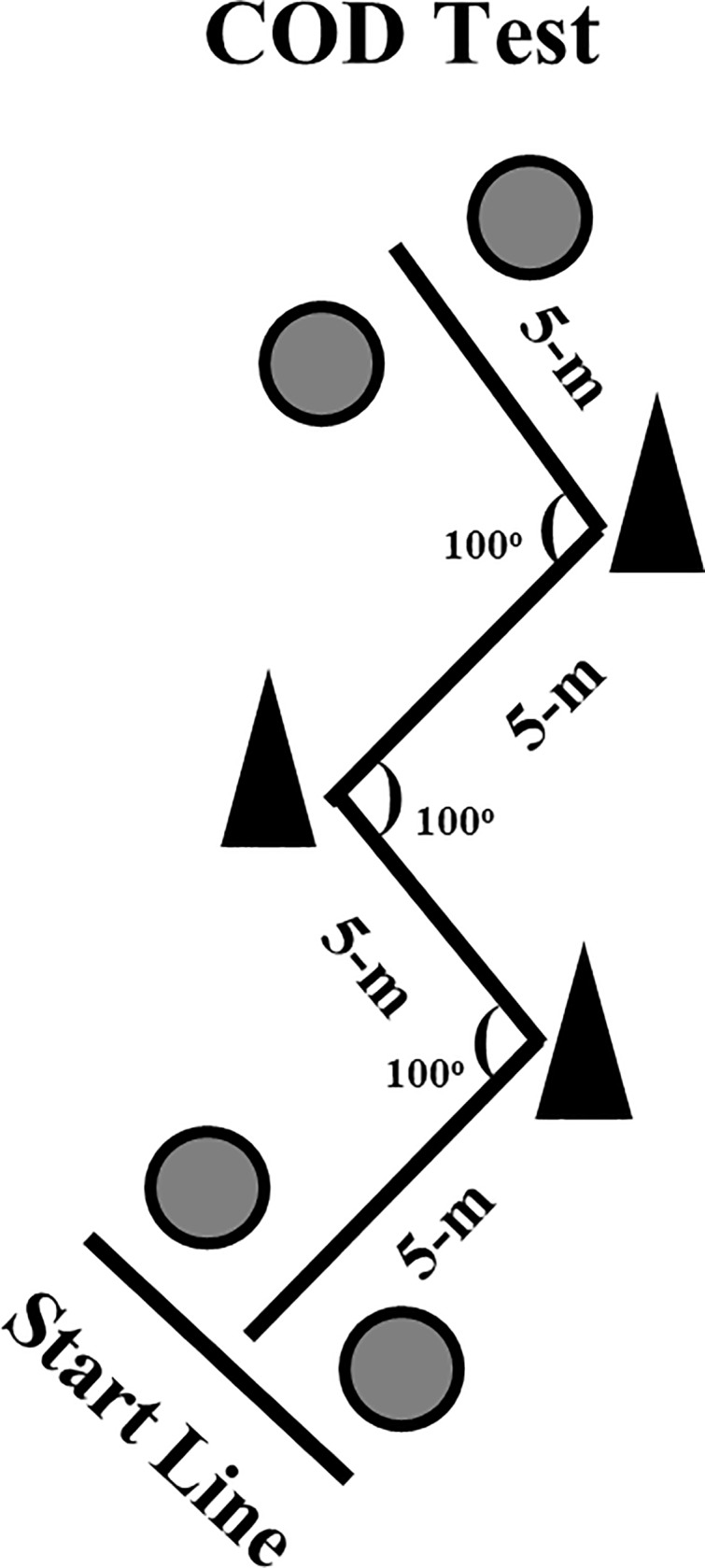
Schematic presentation of the Zig-zag test. Circles represent the position of the photocells.

The COD test was performed on an indoor court and consisted of four 5-m sections (a total of 20-m of linear sprint) marked with cones set at 100° angles (i.e., Zig-zag test, Fig 1), requiring the athletes to decelerate and accelerate as fast as possible around each cone. Two maximal attempts were performed with a 5-min rest interval between attempts. Starting from a standing position with the front foot placed 0.3-m behind the first pair of timing gates (Smart Speed, Fusion Equipment, Brisbane, Australia) (i.e., starting line), the players were instructed to complete the test as quickly as possible, until crossing the second pair of timing gates, placed 20-m from the starting line [10]. The fastest time from the two attempts was retained for further analysis. To evaluate the efficacy of each athlete’s ability to utilize their linear speed during a specific COD task, an adapted COD deficit calculation was used, as described elsewhere [23,25]. Thus, the COD deficit was calculated as follows: *20-m velocity–COD test velocity*.

### Bar-power outputs in jump squat exercise

Maximum bar-power outputs were assessed in jump squat, all performed on a Smith machine (Hammer Strength Equipment, Rosemont, IL, USA). Participants were instructed to execute three repetitions at maximal velocity for each load, starting at 40% of their body mass (BM) in both exercises. Participants executed knee flexion until the thigh was parallel to the ground and, after the command to start, jumped as fast as possible without their shoulders losing contact with the bar. A load of 10% of BM was progressively added for each set until a clear decrement in mean power (MP), mean propulsive power (MPP), and peak power (PP) was observed [35]. A 5-min rest period occurred between sets. To determine the power outputs, a linear position transducer (T-Force, Dynamic Measurement System; Ergotech Consulting S.L., Murcia, Spain) was attached to the Smith machine bar and values were automatically derived by the custom-designed software as follows: MP—value calculated during the entire concentric phase of each repetition; MPP—value calculated during the propulsive phase, defined as that portion of the concentric action during which the measured acceleration is greater than acceleration due to gravity; PP—the highest bar-power value registered at a particular instant (1-ms) during the concentric phase [36,37]. The bar position data were sampled at 1,000 Hz. The maximum MP, MPP, and PP values obtained were used for analysis. Values were normalized by dividing the absolute power by the athletes’ body mass (i.e., relative power = W^.^kg^-1^).

### Statistical analysis

The normality of the data was checked using the Shapiro-Wilk test. Due to the normal distribution, data are described as mean and standard deviation (SD). Athletes were divided, using a median split analysis, into two groups according to their ACC 0-5-m outcome (e.g., higher and lower ACC 0-5-m). This analysis was performed to test the selective influence of the acceleration capacity over a short distance (i.e., 5-m) on the ability to repeatedly accelerate and decelerate over the same distance during a COD task. Magnitude-based inference [38] was used to compare the differences in the physical test results between “higher” and “lower” ACC groups. The quantitative chances of finding differences in the variables tested were assessed qualitatively as follows: <1%, almost certainly not; 1 to 5%, very unlikely; 5 to 25%, unlikely; 25 to 75%, possible; 75 to 95%, likely; 95 to 99%, very likely; >99%, almost certain[38]. If the chances of having better and poorer results were both >5%, the true difference was rated as unclear. The standardized differences for the comparisons in all variables were analyzed using the Cohen’s *d* effect size (ES). The magnitude of the ESs was qualitatively interpreted using the following thresholds: <0.2, trivial; 0.2–0.6, small; 0.6–1.2, moderate; 1.2–2.0, large; 2.0–4.0, very large and; >4.0, nearly perfect [39]. All tests used herein presented good levels of absolute and relative reliability (coefficient of variation <5% and intraclass correlation coefficient >0.90 for all assessments) [39].

## Results

**Fig 2:**
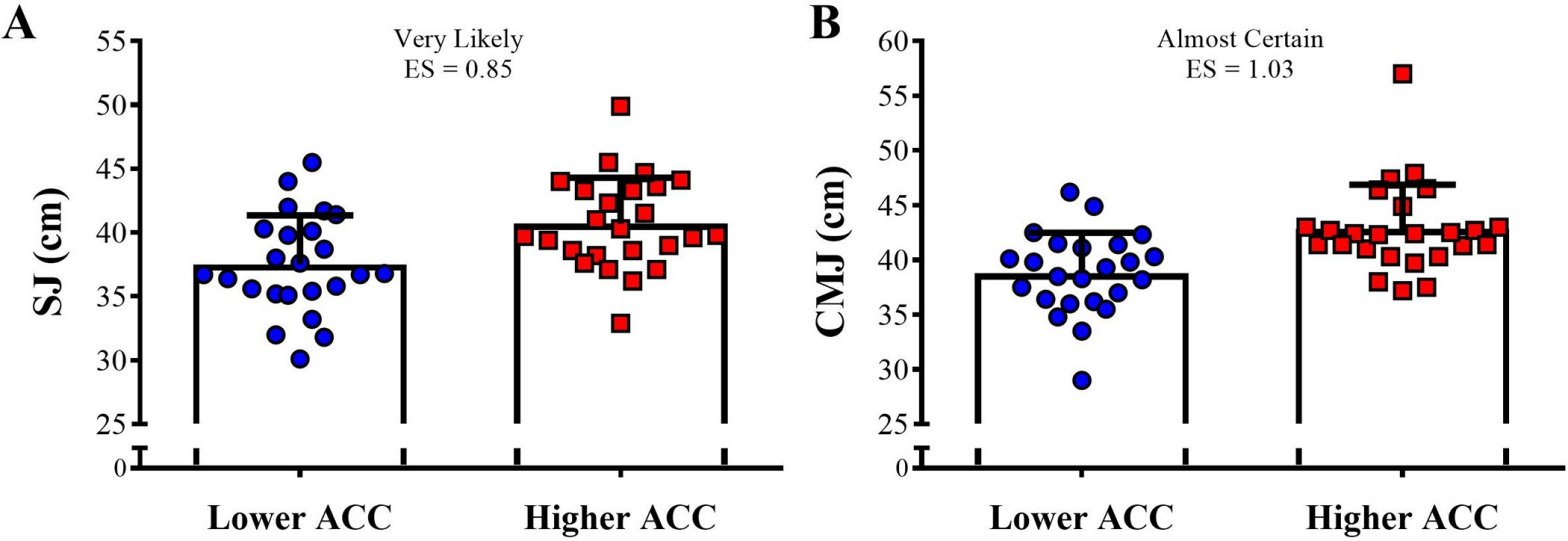
Comparison of the squat and countermovement jumps (SJ and CMJ) between higher and lower acceleration (ACC) groups.

**Fig 3:**
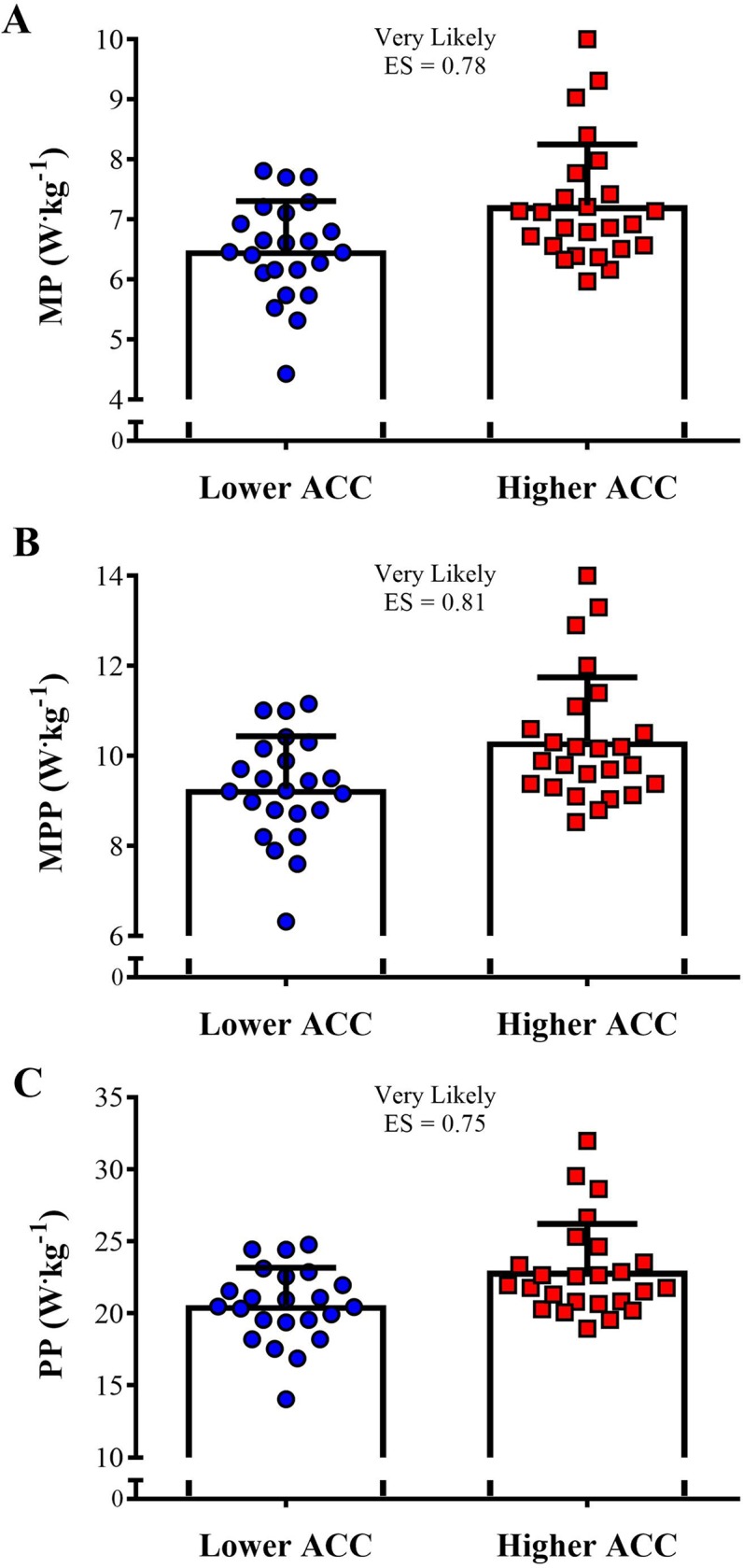
Comparison of the bar-power outputs in the jump squat exercise between higher and lower acceleration (ACC) groups. MP: mean power; MPP: mean propulsive power; PP: peak power.

The ACC 0-5-m in the higher group was almost certainly higher than the lower ACC group (4.98 ± 0.26 m^.^s^-2^ vs. 4.39 ± 0.23 m^.^s^-2^, respectively; ES = 2.37). Fig 2 depicts the comparison of the vertical jumps between higher and lower ACC groups. Very likely and almost certain greater jump heights were observed in the higher ACC group in comparison to the lower ACC group for SJ (ES = 0.85) and CMJ (ES = 1.03), respectively. The comparison of the bar-power outputs in the jump squat exercise between higher and lower ACC groups is demonstrated in Fig 3. The higher ACC group demonstrated very likely grater bar-power outputs than the lower ACC group (ES varying from 0.75 to 0.81).

**Fig 4:**
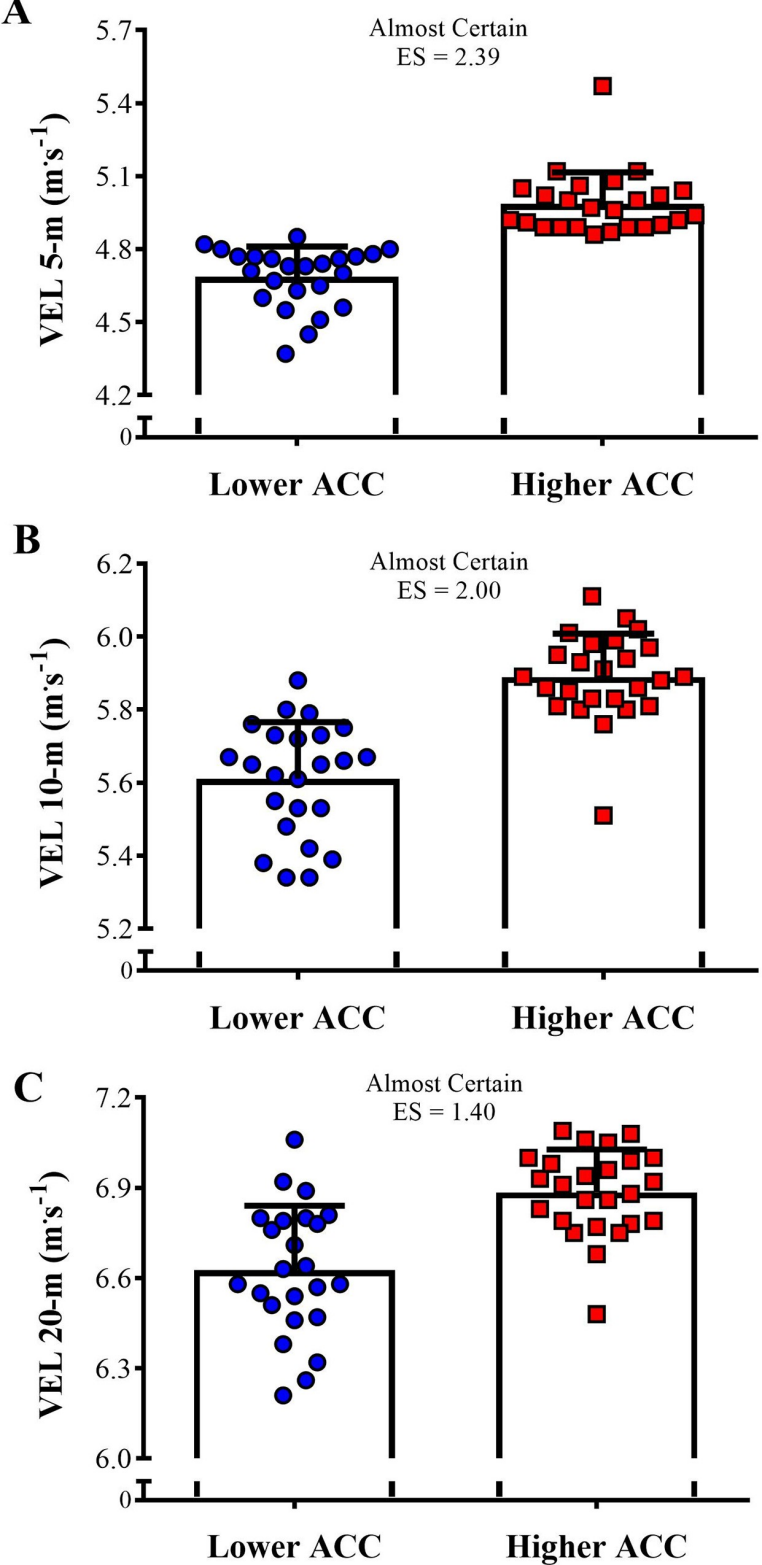
Comparison of the linear sprint velocity in 5-, 10-, and 20-m between higher and lower acceleration (ACC) groups.

**Fig 5:**
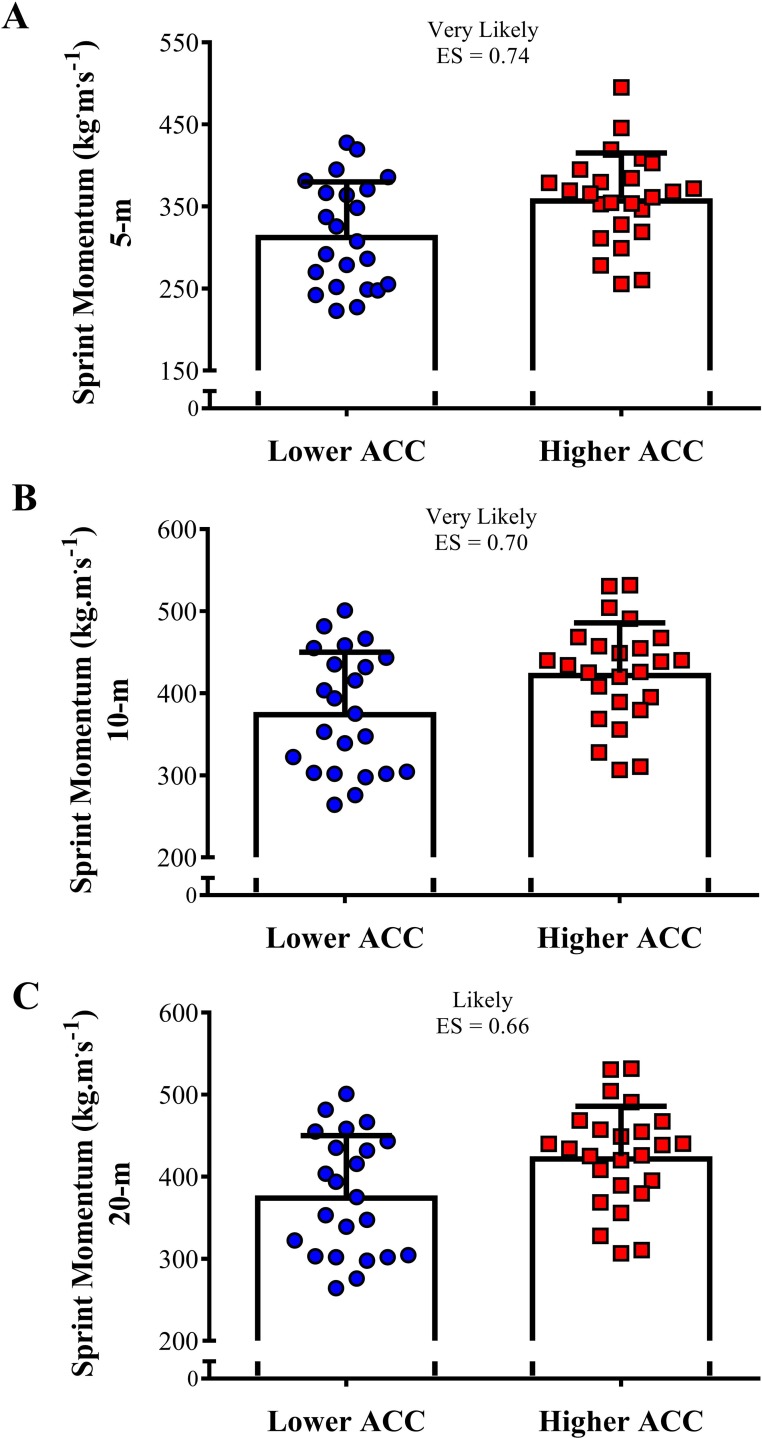
Comparison of the sprint momentum in 5-, 10-, and 20-m between higher and lower acceleration (ACC) groups.

**Fig 6:**
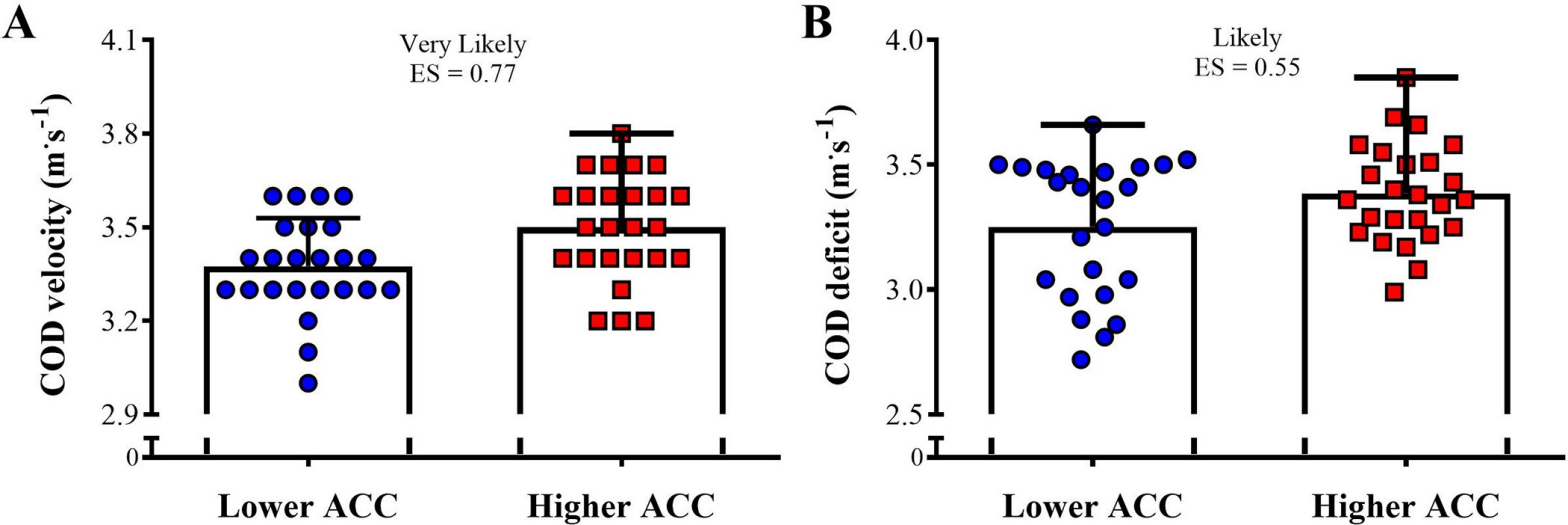
Comparison of the change of direction (COD) velocity and COD deficit between higher and lower acceleration (ACC) groups.

Fig 4 shows the comparison of the linear sprint velocity in 5-, 10-, and 20-m between both groups. The higher ACC group was almost certain faster than the lower ACC group in all distances tested (ES varying between 1.40 and 2.39). Fig 5 demonstrates the comparison of the sprint momentum in 5-, 10-, and 20-m between higher and lower ACC groups. Likely to very likely greater sprint momentum was observed in the higher ACC group in comparison to the lower ACC group in all distances tested (ES varying from 0.66 to 0.74). The comparison of the COD velocity and COD deficit between higher and lower ACC 0-5-m groups is shown in Fig 6. A very likely higher COD velocity was observed in the higher ACC group in comparison to the lower ACC group (ES = 0.77). In addition, the higher ACC group demonstrated a likely greater COD deficit in relation to the lower ACC group (ES = 0.55).

## Discussion

This study examined the selective influences of maximum acceleration capability on COD speed, COD deficit, linear sprint speed, sprint momentum, and loaded and unloaded vertical jump performances in elite soccer players. At first, we observed that soccer players able to accelerate faster over very-short distances (i.e., 0-5-m) were also able to perform better in multiple speed and power-related measurements. Nevertheless, as hypothesized, greater acceleration rates were directly connected with higher COD deficits. To our knowledge, this is the first work to report this important and problematic association in professional soccer players.

To reach higher speeds in short periods of time athletes necessarily have to be able to effectively accelerate over very-short distances (e.g., 5-m). Thus, it is expected that players with higher maximum acceleration rates will be more likely to achieve superior performances in linear sprints up to 20-m (Fig 4). Still in this context, to accelerate more rapidly, they have to apply substantial amounts of force against the ground to overcome the total moment of inertia [31,32,40,41]. Although several investigations have demonstrated the central role played by horizontal force application in sprinting, a consistent body of research also indicates that vertical force production may significantly impact the speed performance of soccer players [32,41,42]. In this regard, a recent study by Colyer et al. [43] revealed that, when compared to sprinters, the limits of the maximum velocities reached by soccer players appear to be related to their lower capacity to generate the vertical impulse required to produce adequate flight times during the acceleration phase of sprinting. The same holds true for vertical jump performance, which has been shown to be significantly associated with the ability to rapidly generate force and power in the vertical direction [31,41,44]. To some extent, these mechanical similarities support the existence of the close correlations typically found between speed and power measures, a fact which has been confirmed in numerous studies involving team sport athletes [30–32,44]. Together, these data may explain why players with greater acceleration rates are more prone to perform better than their slower peers in different speed and power-related tasks (Figs 2, 3 and 4).

Interestingly, as previously reported in other team sports (e.g., rugby union and handball) and younger soccer-categories (using the same COD test employed herein) [16,25,26], the “faster” soccer players over very-short distances also presented higher COD deficits (Fig 6). This suggests that these athletes may be less efficient at coping with their higher approaching velocities, which necessarily increase sprint momentum and, therefore, inertia [26]. It is worth noting that greater momentum is normally associated with higher braking and propulsive forces during sequential decelerations and accelerations, and longer ground contact times in COD drills [20,26]. These “biomechanical compensations” will possibly affect the entry and exit velocities during successive COD maneuvers (especially those with sharp angles; e.g., 100°), reducing the efficiency of faster (and, in this case, more powerful) athletes to change direction [20,26]. As a consequence, despite their superior performance in COD speed tests, professional soccer players with higher maximum acceleration rates tend to lose more time (in relation to their linear sprint performance) when changing direction. However, it is conceivable that COD deficits in drills with less aggressive angles in which velocity maintenance is key (e.g., 45° cutting actions) [20] may be somehow lower even in players with high acceleration rates. This highlights the importance of considering match-specific movement patterns (i.e., the most frequent angles of directional changes according to position or in-match role) before implementing more comprehensive training approaches to adequately develop COD ability in team sport athletes, especially those comprising eccentric exercises, acceleration and deceleration efforts, and specific COD drills [16,25,26,45]. The regular use of these training practices will possibly enhance the players’ ability to tolerate higher approaching velocities, improving their capacity to sequentially accelerate, decelerate, and re-accelerate, which seems to be essential to improve COD efficiency (and reduce COD deficit) [46–48].

In summary, the present results are in line with those previously reported in the literature in other investigations on team sport athletes from different disciplines and age-categories [16,25,26]. From a more general perspective, we observed that soccer players with higher maximum acceleration rates are equally able to jump higher, sprint faster (over short distances), and achieve higher COD speeds than their slower counterparts. Nonetheless, importantly, they appear to be less efficient at changing direction, which may be related to their reduced ability to deal with greater entry and exit velocities [16,20], or counterbalance the associated mechanical consequences (i.e., greater inertia) of being faster and more powerful. This study is inherently limited by its cross-sectional design that does not allow for causal inferences and by the absence of a control group. In addition, we did not implement any type of biomechanical analysis (e.g., foot contact times and peak flexion angles of plant leg and push-off leg when changing direction), which could help us to better understand and explain the technical differences between the faster and slower athletes. Nevertheless, this is the first study to show this problematic association (i.e., higher acceleration rates connected with higher COD deficits) in professional soccer players. These findings may help coaches and researchers to create better and more effective training strategies to improve COD performance in this respective population.

## Conclusions

Professional soccer players with higher maximum acceleration rates tend to perform better in loaded and unloaded jumps and be faster in linear and multidirectional speed tests. On the other hand, as observed in diverse team sport athletes, they also present higher COD deficits. In practical terms, this means that they “spend more time” changing direction than their slower peers, when considering the COD deficit calculation. At the present time we cannot confirm if this paradoxical phenomenon is more related to the mechanical consequences of being faster (i.e., greater inertia) or to technical issues (i.e., biomechanical adjustments), possibly caused by the overutilization of inadequate and ineffective speed training practices (e.g., conventional sprint, strength, and power training sessions). Additional studies should be conducted to examine whether more mixed training approaches (e.g., circuit-training sessions involving eccentric exercises, plyometrics, and successive acceleration-deceleration drills) are capable of improving the COD efficiency in professional soccer players or even to determine if there is an “optimal balance” to be struck between linear speed and COD speed.
